# Untargeted Metabolomic Profile for the Detection of Prostate Carcinoma—Preliminary Results from PARAFAC2 and PLS–DA Models

**DOI:** 10.3390/molecules24173063

**Published:** 2019-08-22

**Authors:** Eleonora Amante, Alberto Salomone, Eugenio Alladio, Marco Vincenti, Francesco Porpiglia, Rasmus Bro

**Affiliations:** 1 Dipartimento di Chimica, Università degli Studi di Torino, Via P. Giuria 7, 10125 Torino, Italy; 2 Centro Regionale Antidoping e di Tossicologia “A. Bertinaria”, Regione Gonzole 10/1, 10043 Orbassano, Italy; 3 Division of Urology, San Luigi Gonzaga Hospital and University of Torino, 10043 Orbassano, Italy; 4 Department of Food Science, Faculty of Science, University of Copenhagen, Rolighedsvej 30, 1958 Frederiksberg, Denmark

**Keywords:** untargeted metabolomics, PARAFAC2, alignment, gas chromatography–mass spectrometry (GC–MS), prostate carcinoma

## Abstract

Prostate-specific antigen (PSA) is the main biomarker for the screening of prostate cancer (PCa), which has a high sensibility (higher than 80%) that is negatively offset by its poor specificity (only 30%, with the European cut-off of 4 ng/mL). This generates a large number of useless biopsies, involving both risks for the patients and costs for the national healthcare systems. Consequently, efforts were recently made to discover new biomarkers useful for PCa screening, including our proposal of interpreting a multi-parametric urinary steroidal profile with multivariate statistics. This approach has been expanded to investigate new alleged biomarkers by the application of untargeted urinary metabolomics. Urine samples from 91 patients (43 affected by PCa; 48 by benign hyperplasia) were deconjugated, extracted in both basic and acidic conditions, derivatized with different reagents, and analyzed with different gas chromatographic columns. Three-dimensional data were obtained from full-scan electron impact mass spectra. The PARADISe software, coupled with NIST libraries, was employed for the computation of PARAFAC2 models, the extraction of the significative components (alleged biomarkers), and the generation of a semiquantitative dataset. After variables selection, a partial least squares–discriminant analysis classification model was built, yielding promising performances. The selected biomarkers need further validation, possibly involving, yet again, a targeted approach.

## 1. Introduction

Prostate cancer (PCa) is the most common non-skin cancer in men [1,2] and the second most frequently diagnosed malignancy in males worldwide [3]. The first biomarker for PCa detection was prostatic acid phosphatase (PAP), which was introduced in the 1930s [1]. In the 1980s, PAP was replaced by prostate-specific antigen (PSA) [1,4], a secreted protein encoded by a prostate-specific gene and member of the tissue kallikrein family [1], which is produced almost exclusively in the prostate [5,6]. After the introduction of PSA, more men were diagnosed with PCa, with the majority having the early-stage, clinically indolent form of the disease. However, a large number of patients affected by a benign pathology, such as inflammation or hyperplasia, exhibited abnormal PSA values, which lead to the execution of useless biopsies and demonstrate the low specificity of this biomarker [1,6]. This phenomenon was generally designated as “overdiagnosis” or “overtreatment” [1,4,7,8].

Considerable effort has been devoted to improving the PSA-test performance, including the introduction of PSA density, PSA velocity (and doubling time), the dosage of free or complexed PSA, and the quantitation of its isoforms [1,2]. A combination of these parameters yields the Prostate Health Index (PHI) [3].

Meanwhile, intensive research has been devoted to the search for different biomarkers, mainly by applying omics methods (e.g., genomics, proteomics, transcriptomics, and metabolomics) [1], and several authors have reviewed the emerging biomarkers, among which the most prominent are the urinary prostate cancer antigen 3 (PCA3) [1,3,5] and transmembrane protease, serine 2 (TMPRSS2-ERG) (sometimes combined together) [1,3,5]. Alpha-methylacyl-CoA Racemase (AMACR) demonstrated high sensitivities and specificities on prostate biopsy, but it is not suitable for non-invasive detection in urine [1,5]. Increased diagnostic performances were obtained by the serum dosage of human kallikrein-related peptidase 2 (KLK2) in combination with total and free PSA [5]. An evolution of the application of kallikreins consists in a blood measurement of the four existing isoforms which, combined with clinical information, allows the probability calculation of PCa incidence [3]. Significantly increased levels of prostasomes were found in blood samples from patients with PCa [9], while elevated levels of urinary sarcosine were found to be associated with aggressive forms of prostate cancer [1].

Studies conducted in the 1970s and 1980s highlighted the correlation between increased urinary excretion of polyamines (i.e., spermine, spermidine, and putrescine) and several types of cancer [10,11]. However, anomalous oxidative degradation reactions of these polyamines resulted in low concentrations of these biomarkers in approximately 20% of the patients, leading to false-negative prediction and consequently limiting their application as diagnostic biomarkers [10].

The correlation between altered steroidal biosynthesis and PCa is well known [12,13,14]. For this reason, in a previous study, we carried out a targeted analysis of urine samples, addressed to a large panel of androgens, including testosterone and its principal phase I metabolites. The multivariate statistical interpretation of these steroid profiles produced satisfactory results in terms of sensitivity, specificity, and area under the curve (AUC) [15]. 

In this study, the search for new urinary biomarkers was undertaken by using untargeted methods. In perspective, emerging biomarkers could possibly be combined with the most discriminating steroid biomarkers to improve their screening performances further, without altering the inherent simplicity of the instrumental procedure. In fact, the ideal biomarker should be cheap to determine, non-invasive, easily accessible, and quickly quantifiable [1,2]. Taking into account the abovementioned considerations, gas chromatography–electron impact mass spectrometry (GC–EIMS) would give a more suitable solution than the other commonly used analytical techniques to provide a three-dimensional pattern for untargeted analysis. Urine was chosen as the election matrix, as it is easily available in large volumes and involves non-invasive sampling.

## 2. Materials and Methods

### 2.1. Chemicals and Reagents

Tert-butyl methyl ether (TBME), ethyl acetate, dithioerythritol, ammonium iodide (NH_4_I), *N*-Methyl-*N*-(trimethylsilyl)trifluoroacetamide (TMSTFA), and trifluoroacetic anhydride (TFAA) were provided by Sigma-Aldrich (Milan, Italy). β-glucuronidase from *Escherichia coli* was purchased from Roche Life Science (Indianapolis, IN, USA). Ultra-pure water was obtained using a Milli-Q^®^ UF-Plus apparatus (Millipore, Bedford, MA, USA). 

### 2.2. Samples Collection

The subjects involved in this study were recruited in the ambulatory of the Department of Urology at the San Luigi Hospital of Orbassano (TO, Italy), after approval of the protocol by the reference Ethical Committee (protocol number 0019267). A total of 91 subjects were enrolled, including 43 affected by prostate carcinoma (PCa, confirmed by a positive biopsy) and 48 diagnosed with benign prostatic hyperplasia (BPH, with a PSA lower than the European cut-off of 4 ng/mL or with a PSA above the threshold but a negative biopsy result). In a previous study, the progressive modification of the urinary steroidal profile with age was investigated [16]. From this study, we decided to enroll only individuals older than 60 years, when the bias effect due to aging became negligible [16]. Moreover, since ethnicity represents another important bias factor, only Caucasian individuals were recruited. Finally, diabetes, other carcinoma, metabolic diseases, and therapies suspected to alter the urinary steroid profile (such as steroid therapy) were considered as exclusion criteria.

Body mass index (BMI), alcohol consumption, medical therapy, digital rectal examination, PSA value, and biopsy Gleason Score (GS) were recorded. In detail, the group’s mean age and standard deviation was 70 ± 10 years for BPH and 70 ± 8 years for PCa. BMI was within the range of normality for all individuals (between 18.5 and 25), and PSA was 3.8 ± 2.3 ng/mL for BPH and 11.0 ± 9.5 ng/mL for PCa. The PCa class was distributed as low risk (GS = 3 + 3, 15 patients), middle risk (GS = 3 + 4 and 4 + 3, 21 patients), and high risk (GS = 4 + 4 and 4 + 5, seven patients).

### 2.3. Sample Treatment and GC–MS Analysis

Firstly, the protein components of the urinary samples were precipitated by centrifugation at 4000 rpm for 5 min. Two aliquots (A and B) of 5 mL each were taken from each sample. The urine pH was adjusted between 6.8 and 7.4 by adding 2 mL of phosphate buffer and a few drops of NaOH 1M or HCl 1M whenever necessary. Enzymatic hydrolysis of the glucuronide metabolites was conducted with 100 µL of β-glucuronidase from *Escherichia coli* (equivalent to 83 enzymatic units) by heating it in the oven for 1 h. After cooling to room temperature, the two aliquots were subjected to different liquid–liquid extraction (LLE) with 5 mL of TBME each, at basic (pH ≥ 10) and acid (pH ≤ 1) conditions, respectively, obtained by the introduction of some drops of NaOH 1M and HCl 1M. Both aliquots were dried under a gentle nitrogen stream at room temperature. The dried aliquot A was derivatized using 50 µL TFAA at 65 °C for 1 h. Then, the solvent was dried and the residue was dissolved in 50 µL TBME and injected into the GC–MS. The chromatographic separation was achieved with a J&W Scientific HP-5, 17 m × 0.2 mm (i.d.) × 0.33 µm (f.t.) capillary column. The oven temperature was programmed as follows: The starting temperature of 90 °C was held for 1 min. Then, the temperature of 180 °C was reached with a rate of 30 °C/min and held for 7 min. A final heating rate of 15 °C/min was applied until the temperature of 325 °C was reached (held for 3 min). The chromatographic run lasted 22.20 min. 

Aliquot B was derivatized using 50 µL of TMSTFA/NH_4_I/dithioerythritol (1.000:2:4 *v*/*w*/*w*), at 70 °C for 30 min and then injected into a GC–MS equipped with a J&W Scientific HP-1, 17 m × 0.2 mm (i.d.) × 0.11 µm (f.t.) capillary column. The oven temperature was programmed to heat up from 120 to 177 °C at a rate of 70 °C/min, and from 177 to 236 °C at a rate of 5 °C/min. A final heating rate of 30 °C/min was applied until the temperature of 315 °C was reached. The chromatographic run lasted 18.25 min. Both the runs were performed in full-scan mode, in the interval 40–650 *m*/*z* at a scan rate of 2.28 scans/s.

Because the samples were analyzed in five analytical sections performed on different days, it was important to monitor the occurrence of a data structure due to the different analytical sections. The exploratory unsupervised data analysis can serve to this scope, and principal component analysis (PCA) was employed. No clustering or trend related to the day of the analysis was detected.

### 2.4. Statistical Analysis

**Figure 1:**
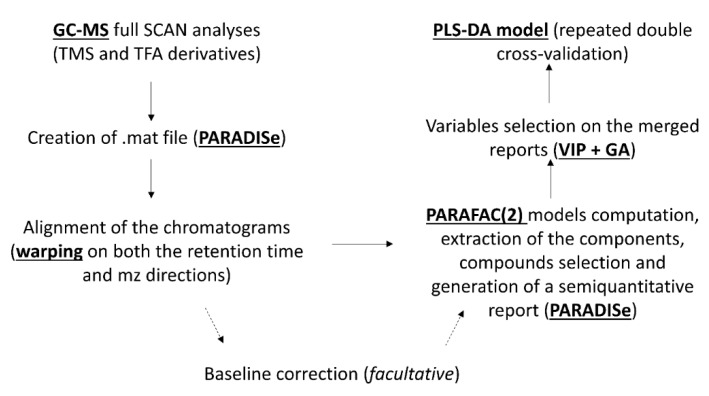
Statistical analysis workflow.

The main steps of the statistical analysis are reported in Figure 1. 

### 2.5. Pre-Treatment of the Raw Data

The .AIA files of the chromatographic runs were downloaded using the software ChemStation^®^.

The PARADISe version 3 software was employed to convert the files in a form suitable for MATLAB (extension .mat). The alignment procedure, both propaedeutic and mandatory for the following steps of data analysis, was executed over the three-way (samples × retention time × *m/z*) array of size 91 × 3099 × 612 (over 172 million data) and 91 × 1640 × 652 (over 97 million data) for the trifluoroacetyl (TFA) and trimethylsilyl (TMS) derivatives, respectively. The correlation optimized warping (COW) was performed along the retention time and the *m*/*z* dimensions [17]. The two matrices were segmented along the retention time dimension to improve the performances of the COW algorithm, and for each slice the computation was iterated until a visually satisfying result was obtained. Lastly, to improve the visualization of the data, the baseline was subtracted. It is important to highlight that the latter computational step only served to improve the data visualization by the operator, because PARAFAC2 is able to recognize the baseline and the noise components, allowing their automatic exclusion [18,19,20,21]. 

### 2.6. PARAFAC2 Models Computation and Molecular Identification

The aligned dataset was analyzed in the PARADISe software, to proceed with the computation of PARAFAC2 models; the operating procedure consists in the manual identification of intervals along the chromatogram (with each interval ideally containing approximately one peak). The PARAFAC2 models were built introducing the non-negativity constraint and performing 10,000 iterations for interval [18,19,20,21]. 

Within the software, the operator can label the components as (i) baseline, (ii) noise, or (iii) compounds. All the mass spectra of the components belonging to the third category are automatically compared with the NIST database. A report is produced, including the relative concentrations of the detected compounds (assuming a uniform response factor of 1), and the *n* (number subjectively chosen by the user) most likely identifications for each compound. Finally, the relative concentrations were normalized using the urinary creatinine values.

### 2.7. Classification Models

The dataset composed by the relative concentrations of the detected metabolites for each sample was used to perform partial least squares–discriminant analysis (PLS–DA) [22], classifying the samples into having prostate carcinoma or not. Firstly, the dataset was log10 transformed (with the aim of achieving a more even distribution of each of the variables) and autoscaled. Then, the variable importance in projection (VIP) method (using a threshold of 1) [23] and genetic algorithms (GAs) [24] were run to select the most relevant variables. The reduced dataset was finally used to build the PLS–DA classification model. The model was then validated using the repeated double cross-validation (dCV) approach [25]. The PLS_Toolbox version 8.5 (Eigenvector Research, Inc., Manson, WA, USA) was used to perform this part of the analysis [26]. 

## 3. Results and Discussion

**Figure 2:**
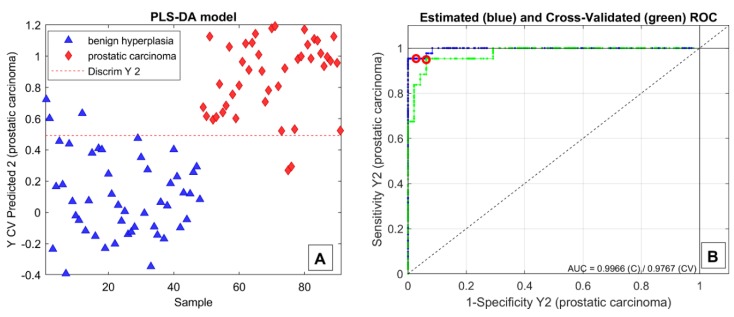
Y-value predicted in cross-validation (CV) (**A**) and receiver operating characteristic (ROC) curves (**B**) of one of the several classification models built during the repeated double cross-validation procedure.

The preliminary PARAFAC2 model extracted a total of 329 relevant compounds (184 from the chromatographic run after TMS derivatization and 145 after TFA derivatization). Of these, 89 were selected using the VIP algorithm, and a further 58 substances were discarded by one cycle of GAs. The final dataset, consisting in a 91 × 32 (subjects × variables) matrix, was employed to build a PLS–DA classification model. Due to the heterogeneity of the patients enrolled (in terms of pathology staging, prostatic volume, and PSA values), the model was validated using repeated double cross-validation (30 repetitions were performed) [25] instead of the standard external validation, in which the use of a limited and heterogeneous population may result in significant bias. The plot reporting the Y-value predicted in cross-validation (CV) in one of the several classification models produced during the repeated double cross-validation process is shown in Figure 2A. The corresponding receiver operating characteristic (ROC) curve is depicted in Figure 2B. The high values of the area under the curve (AUC) for both the estimated and cross-validated ROCs are an indicator of high performances and robustness of the model. In detail, using a discriminating Y-value of 0.5, the model provides 92.5 ± 2.2% sensitivity and 88.7 ± 3.9% specificity for the cancer-affected population. On average, misclassification occurred on about 3 ± 2 patients affected by carcinoma out of 43 and 5 ± 2 patients with hyperplasia out of 48.

Of the 32 compounds selected by the dedicated algorithms to build the model, 17 were not found in the available NIST libraries, while for seven other compounds, the identification provided by automatic spectral matching was deemed incorrect. On the other hand, manual mass spectra interpretation was made difficult by the structural similarity of many candidate biomarkers, as well as the effect of the derivatizations, that introduced functional groups (e.g., −Si(CH_3_)_3_) yielding prevalent fragment ions in the spectrum. The mass spectra of the 32 metabolites are provided as Supplementary Materials (Supplementary Figure S1). Table 1 reports the eight identified compounds, accompanied by their Human Metabolome Database (HMDB) and Kyoto Encyclopedia of Genes and Genomes (KEGG) identification numbers, when available. Since the PARADISe output provides only a rough semiquantitative report based on the total ion current (TIC) without any external calibration, the real physiological concentration of each metabolite could not be evaluated. However, these absolute TIC values can be evaluated in relative terms to provide an averaged qualitative comparison between the two populations for all the analytes. The overexpression and underexpression of these metabolites allegedly linked to the occurrence of PCa are reported in Table 1.

It is interesting to note that among the eight identified compounds, five (63%) are involved in steroidal biosynthesis, confirming their potential in the detection and diagnosis of PCa. Similarly, Choi et al. found elevated levels of 16-hydroxy-dehydroepiandrosterone, epiandrosterone, etiocholanolone, and pregnanetriol in patients diagnosed with papillary thyroid carcinoma [27]. The first steroid appears to also be overexpressed in the present case for patients with PCa, but pregnanetriol was underexpressed in the same patients and epiandrosterone was found in comparable concentrations in the two populations. Dehydroepiandrosterone is involved in the expression of insulin-like growth factor 1, whose dysregulation is implicated in certain colon, liver, prostate, and breast cancers [28]. This observation may justify the inclusion of 16-hydroxy-dehydroepiandrosterone among the potential biomarkers for PCa. Pregnanetriol, together with 5 β-pregnanediol, is also known to be dysregulated in adrenal syndromes, such as adrenal tumors or Cushing’s syndrome [29,30]. Increased androsterone levels were found in a cohort of PCa-affected individuals within a multivariate investigation of the urinary steroidal profile, and the present findings are in accordance with our previous study [16]. Other steroids that proved useful to discriminate PCa from BPH [27] were possibly overlooked in the present untargeted selection because of their low concentration in urine.

The overexpression of serotonin and its biomarkers (among which, 5-hydroxyindoleacetic acid) represents a potential urinary biomarker for neuroblastic and carcinoid tumors [31]. While there is no evidence in the literature of an association between 5-hydroxyindoleacetic acid and PCa, the present data suggest such a hypothesis, as its overexpression is clearly evident in the PCa-affected population considered. 

Phytoestrogens are a class of substances accredited to prevent the onset of cancer [32,33]. In accordance with this hypothesis, enterodiol is underexpressed in the present PCa population.

**Figure 3:**
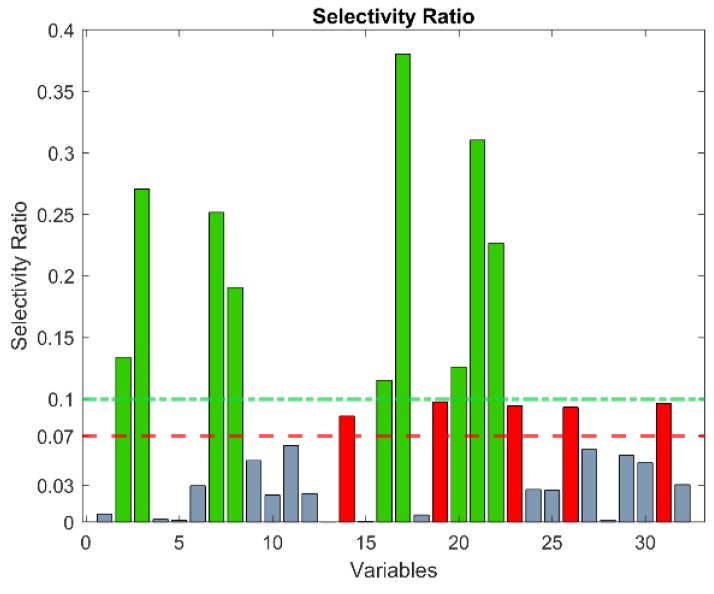
Selectivity ratio of the 32 selected features. The variables above the threshold of 0.1 are reported in green, and the ones between the thresholds 0.07–0.1 are reported in red.

**Figure 4:**
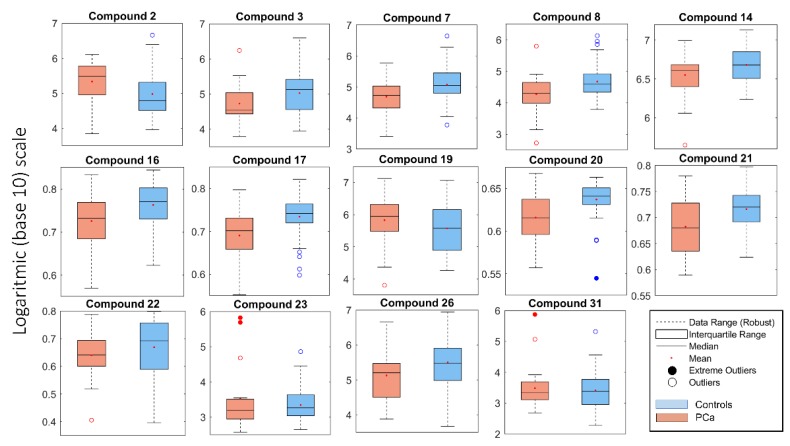
Boxplot, in logarithmic (base-10) scale, of the 14 compounds above the selectivity ratio threshold of 0.07 (see Figure 3).

Among the 32 selected biomarkers, different contributions to the overall discrimination achieved by the PLS–DA model (Figure 1) were expected. A rough estimation of the relative importance of these biomarkers is expressed by their selectivity ratios [34], reported in Figure 3. Nine biomarkers exhibit selectivity ratios higher that 0.1, while, for five others, values between 0.07 and 0.1 were found. Interestingly, out of the nine biomarkers with the highest selectivity ratio, eight are underexpressed in the PCa patients, apparently suggesting them as protective substances. The expression of the 14 metabolites is represented in the form of boxplots in Figure 4. Tentative PLS–DA models were built with only these 9 and 14 biomarkers, but their overall efficiency significantly dropped with respect to the model of 32 biomarkers, demonstrating that the relative contribution of the remaining biomarkers is not negligible. In particular, the specificity index was considerably reduced in the models of 9 biomarkers and 14 biomarkers, while the sensitivity score remained relatively high.

Further testing was also conducted on the six biomarkers showing comparable mean intensity for the two populations. One variable at a time was removed, and a new classification model was computed using a simple cross-validation with each reduced dataset. Five of the seven new models yielded decreased sensitivity and specificity, while the other two models provided comparable performance, substantially confirming the choice of the 32 biomarker model.

## 4. Conclusions

The preliminary results reported in the present study support the premise that GC–MS tridimensional data can be profitably exploited in untargeted metabolomics studies devoted to prostatic carcinoma diagnosis. Compared to the more resource-demanding ultra-high-performance liquid chromatography–tandem mass spectrometer (UHPLC–MS/MS) and ultra-high-performance liquid chromatography–high-resolution mass spectrometry (UHPLC–HRMS) approaches frequently presented in the literature, GC–MS offers comparable chromatographic resolution and structured spectroscopic information, as is generated by the fragment ion pattern typical of electron impact ionization. On these complex data arrays, the ultimate performance in the extraction of crucial information relies on the software purposely adopted and PARAFAC2 combined with VIP and GA methods of variables selection proved to produce highly efficient models of class discrimination, allowing us to distinguish prostatic carcinoma from benign hyperplasia with good sensitivity and specificity scores.

A common limitation of untargeted metabolomics methods, including the present one, is that the most abundant components of the screened samples are preferentially isolated as potential biomarkers with respect to minor constituents, possibly present at trace levels. This explains the differences in the selected biomarkers with respect to the targeted approach that we previously tested [27]. On the other hand, complementary sets of biomarkers are extracted and then evaluated from targeted and untargeted approaches, to be subsequently combined to achieve optimal performance. More work has to be done on large populations of PCa-affected patients and controls to confirm the present findings, and further effort is necessary to reveal the identity of the most valuable biomarkers and possibly confirm their real value as interesting biomarkers by univariate statistics. Despite these limitations to be overcome in the subsequent investigations, the strategy adopted in the present study, based on non-invasive urine sampling, cheap instrumentation, and advanced data treatment by PARADISe software, appears to be extremely promising in PCa screening.

## Figures and Tables

**Table 1:** List of the 32 selected metabolites. The kind of derivatization, retention time, and expression in prostate cancer (PCa)-affected individuals are reported. Moreover, metabolites with a putative identification are accompanied by the match score with NIST library and the relative identification (ID) number in the Human Metabolome Database (HMDB) and the Kyoto Encyclopedia of Genes and Genomes (KEGG) database. The mass spectra of the 32 metabolites are reported in Supplementary Materials—Figure S1.

	Compound		Derivatization	Retention Time (min)	Match with NIST	HMDB ID	KEGG ID	Expression in PCa Patients
**TMS derivatives**	1	5-Hydroxyindoleacetic acid	TMS	5.26	893	HMDB0000763	C05635	overexpression
	2	Unknown 1	TMS	5.86	-	-	-	overexpression
	3	Unknown 2	TMS	7.44	-	-	-	underexpression
	4	Androsterone	TMS	8.14	912	HMDB0000031	C00523	overexpression
	5	16-Hydroxydehydroisoandrosterone	TMS	9.23	888	HMDB0000352	C05139	overexpression
	6	Unknown 3	TMS	9.84	-	-	-	comparable
	7	Unknown 4	TMS	10.31	-	-	-	underexpression
	8	Unknown 5	TMS	10.61	-	-	-	underexpression
	9	Unknown 6	TMS	11.29	-	-	-	underexpression
	10	Unknown 7	TMS	11.32	-	-	-	comparable
	11	Enterodiol	TMS	12.19	826	HMDB0005056	C18166	underexpression
	12	5β-pregnanediol	TMS	12.53	853	HMDB0005943	Not available	underexpression
	13	Unknown 8	TMS	13.6	-	-	-	overexpression
	14	Unknown 9	TMS	13.67	-	-	-	comparable
	15	Pregnanetriol	TMS	13.73	904	HMDB0006070	Not available	underexpression
	16	Unknown 10	TMS	14.03	-	-	-	underexpression
	17	Unknown 11	TMS	14.50	-	-	-	underexpression
	18	Unknown 12	TMS	14.53	-	-	-	underexpression
	19	Unknown 13	TMS	14.6	-	-	-	overexpression
	20	Unknown 14	TMS	14.66	-	-	-	underexpression
	21	Unknown 15	TMS	15.04	-	-	-	underexpression
**TFA derivatives**	22	Unknown 16	TFA	1.63	-	-	-	underexpression
	23	Unknown 17	TFA	1.71	-	-	-	comparable
	24	Vanillyl alcohol	TFA	3.37	860	HMDB0032012	C06317	overexpression
	25	Unknown 18	TFA	4.97	-	-	-	comparable
	26	Unknown 19	TFA	5.71	-	-	-	underexpression
	27	Unknown 20	TFA	3.32	-	-	-	underexpression
	28	Epiandrosterone	TFA	15.61	925	HMDB0000365	C07635	comparable
	29	Unknown 21	TFA	16.32	-	-	-	underexpression
	30	Unknown 22	TFA	17.87	-	-	-	underexpression
	31	Unknown 23	TFA	18.11	-	-	-	overexpression
	32	Unknown 24	TFA	18.24	-	-	-	underexpression

## Supplementary Materials

The following are available online.
