# Supplementary material for: Untargeted Metabolomic Profile for the Detection of Prostate Carcinoma—Preliminary Results from PARAFAC2 and PLS–DA Models
Source: Molecules. 2019 Aug 22;24(17):3063. doi: 10.3390/molecules24173063 (PMC6749415; doi:10.3390/molecules24173063)
Supplement: Supplementary file 1 [file molecules-24-03063-s001.pdf]

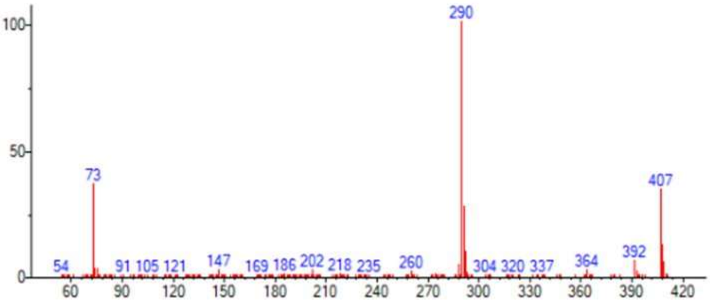

5-Hydroxyindoleacetic acid (TMS tri-derivative)

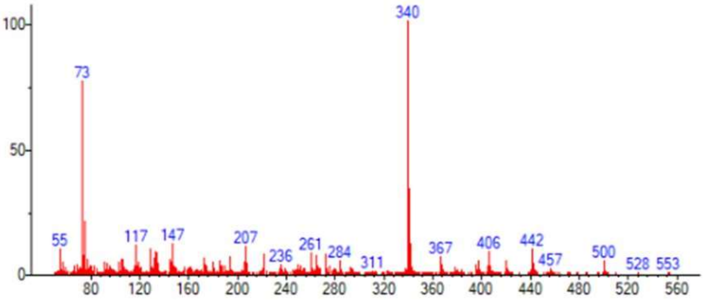

Unknown 1 (TMS derivative)

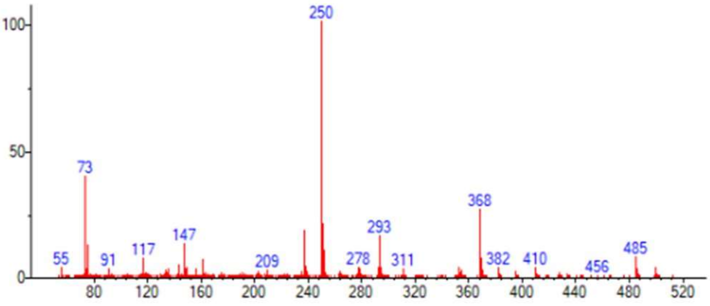

Unknown 2 (TMS derivative)

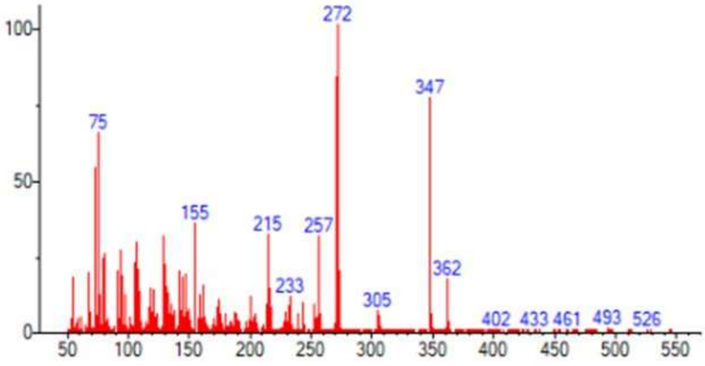

Androsterone (TMS derivative)

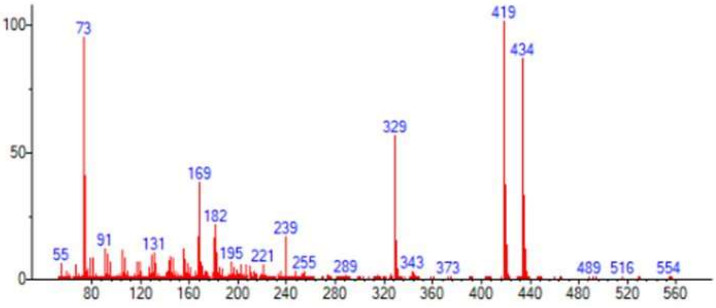

16-Hydroxydehydroisoandrosterone (TMS di-derivative)

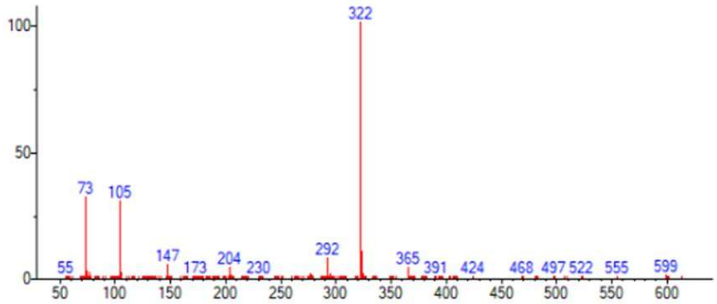

Unknown 3 (TMS derivative)

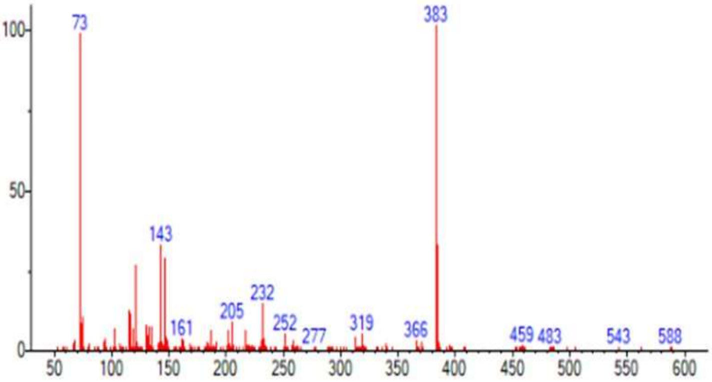

Unknown 4 (TMS derivative)

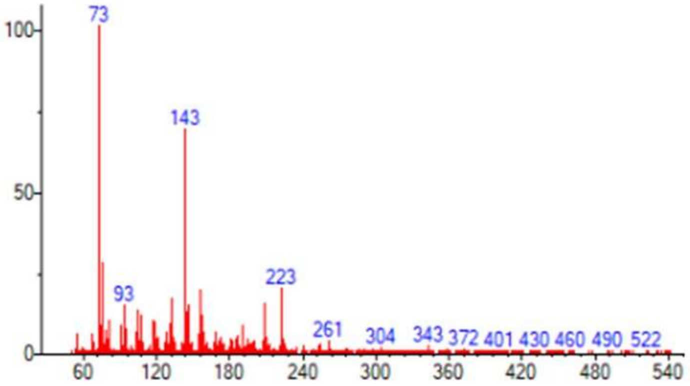

Unknown 5 (TMS derivative)

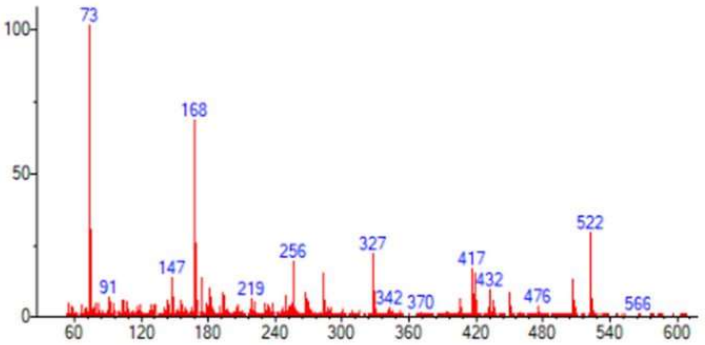

Unknown 6 (TMS derivative)

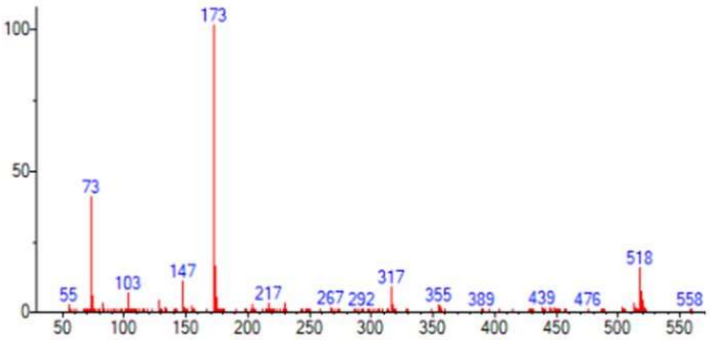

Unknown 7 (TMS derivative)

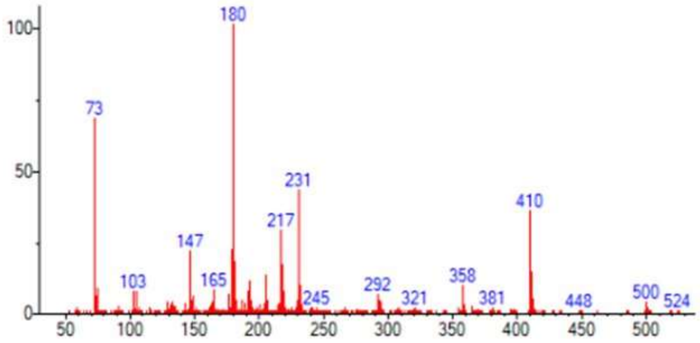

Enterodiol (TMS tetra-derivative)

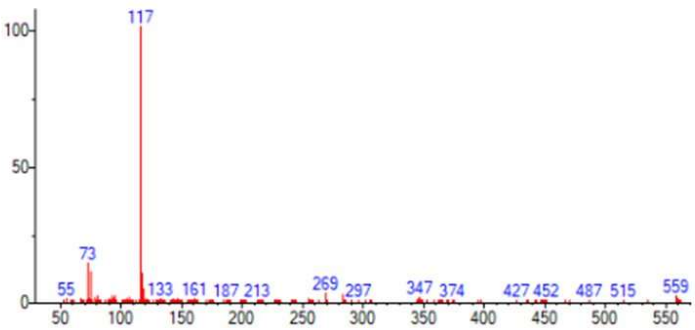

5-Pregnanediol (TMS di-derivative)

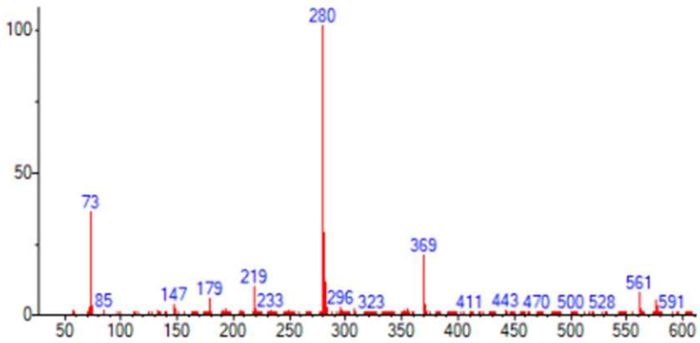

Unknown 8 (TMS derivative)

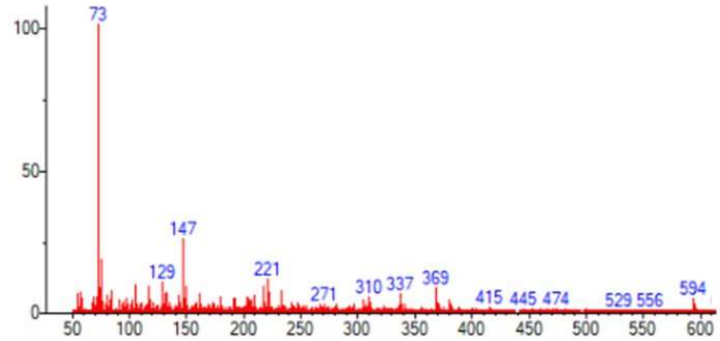

Unknown 9 (TMS derivative)

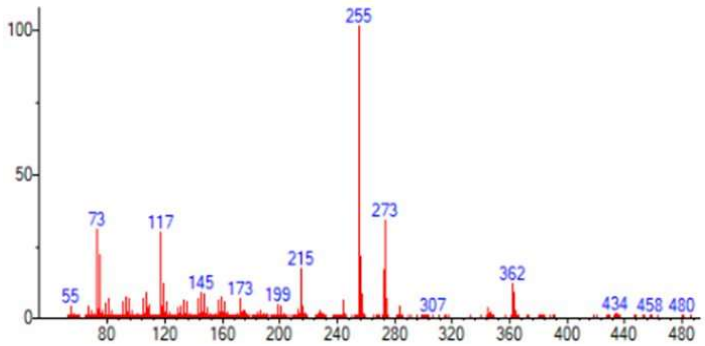

Pregnanetriol (TMS di-derivative)

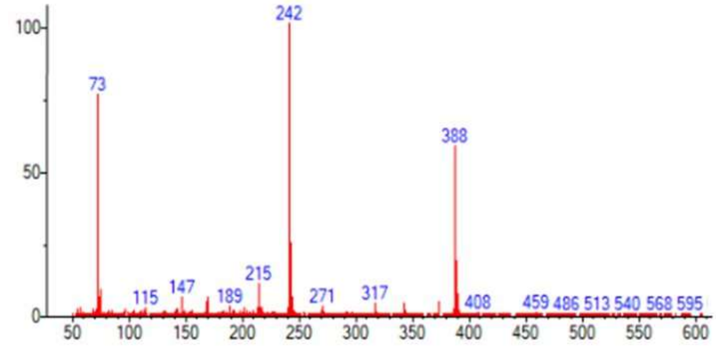

Unknown 10 (TMS derivative)

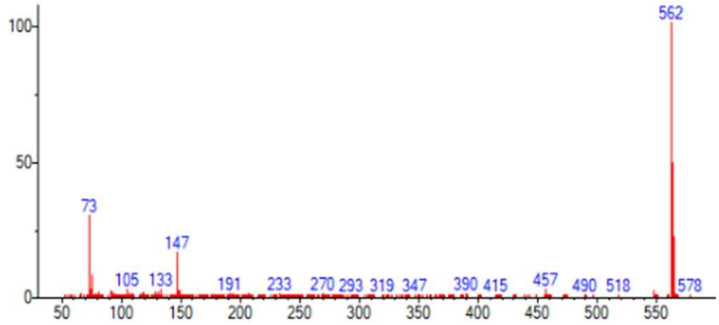

Unknown 11 (TMS derivative)

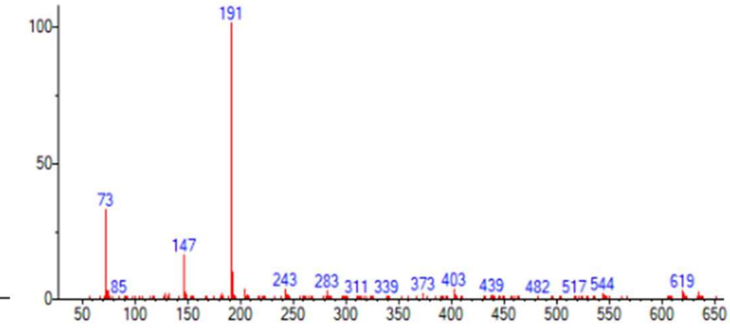

Unknown 12 (TMS derivative)

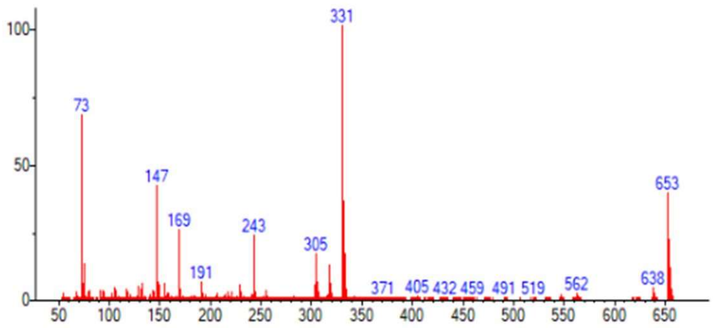

Unknown 13 (TMS derivative)

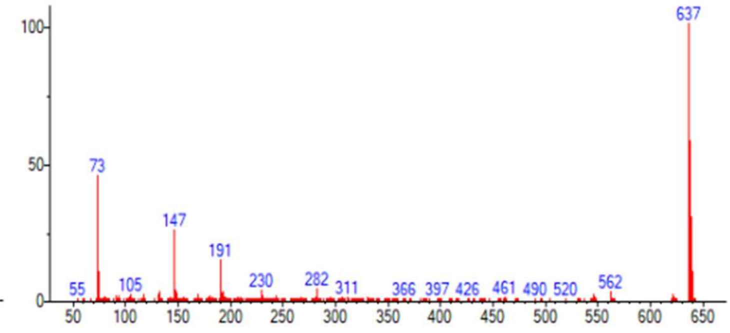

Unknown 14 (TMS derivative)

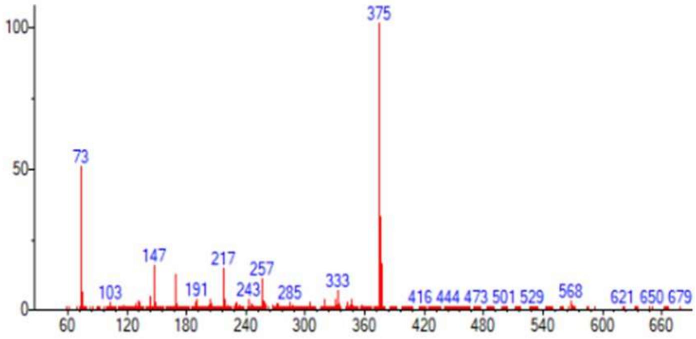

Unknown 15 (TMS derivative)

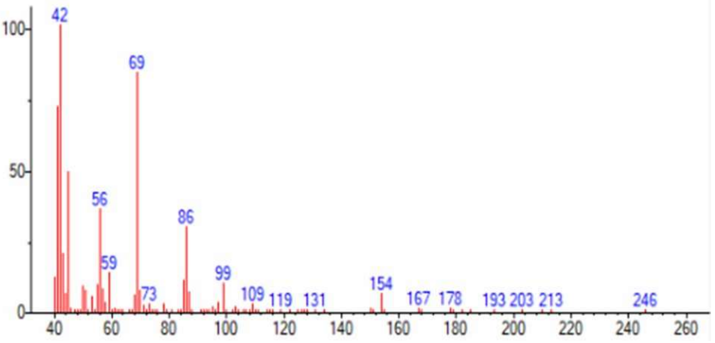

Unknown 16 (TFA derivative)

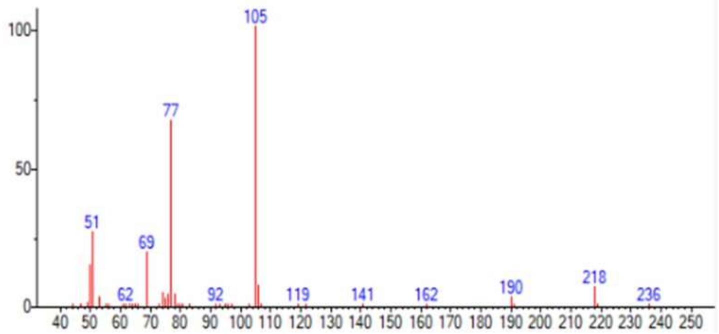

Unknown 17 (TFA derivative)

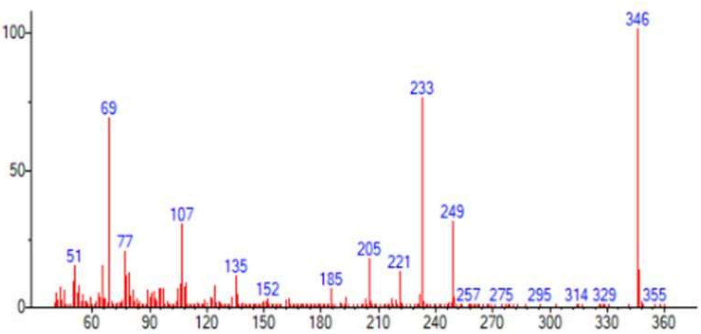

Vanillyl alcohol (TFA di-derivative)

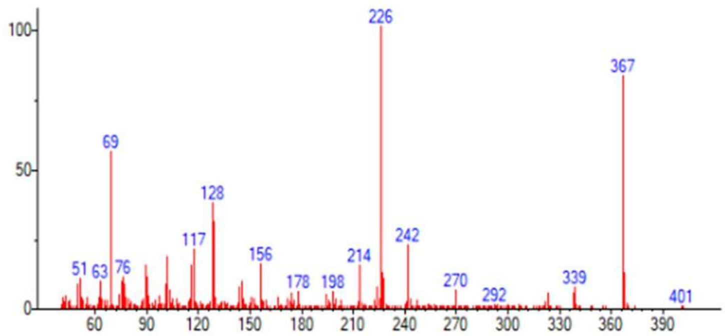

Unknown 18 (TFA derivative)

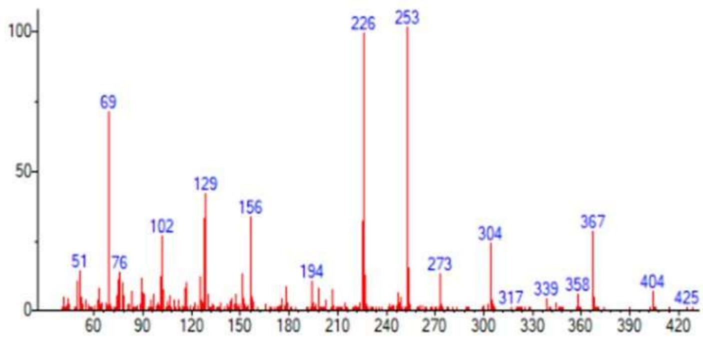

Unknown 19 (TFA derivative)

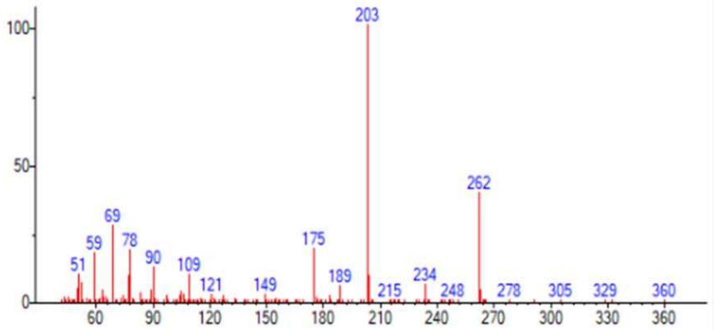

Unknown 20 (TFA derivative)

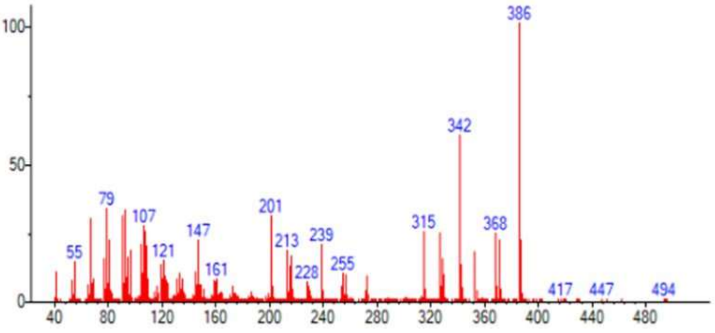

Epiandrosterone (TFA derivative)

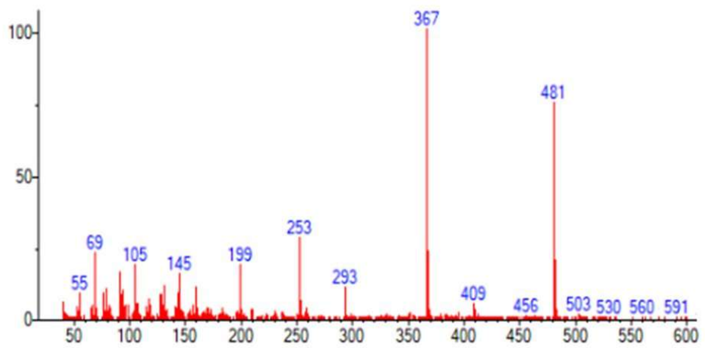

Unknown 21 (TFA derivative)

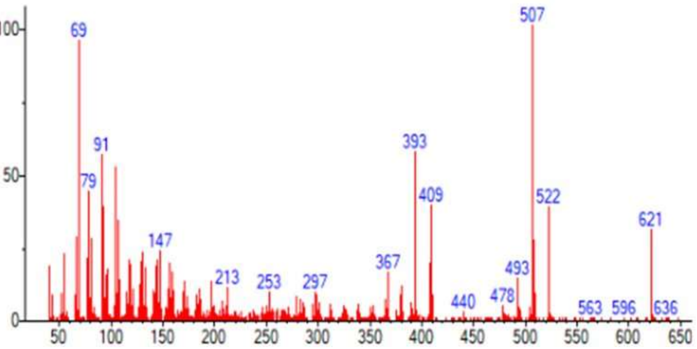

Unknown 22 (TFA derivative)

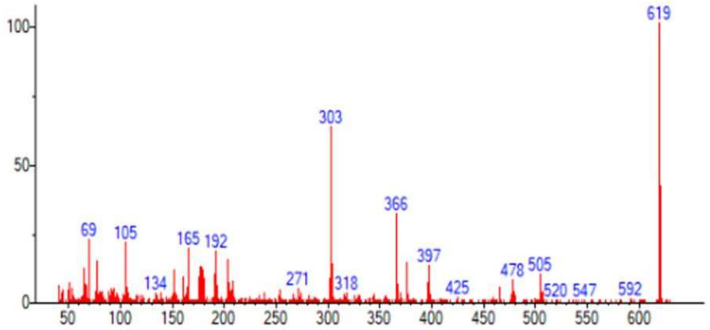

Unknown 23 (TFA derivative)

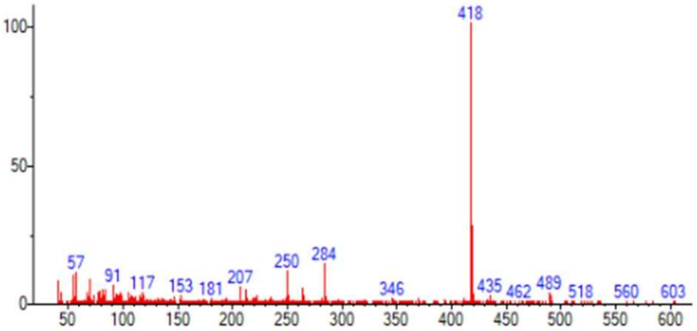

Unknown 24 (TFA derivative)

Supplementary Figure S1. EI mass spectra of the 32 selected metabolites, derivatized with either TMS (up to "Unknown 15") or TFA (from "Unknown 16" to "Unknown 24").
